# The effect of pregnancy on growth-dynamics of neurofibromas in Neurofibromatosis type 1

**DOI:** 10.1371/journal.pone.0232031

**Published:** 2020-04-28

**Authors:** Lennart Well, Anna Jaeger, Hildegard Kehrer-Sawatzki, Said Farschtschi, Maxim Avanesov, Markus Sauer, Manuela Tavares de Sousa, Peter Bannas, Thorsten Derlin, Gerhard Adam, Victor F. Mautner, Johannes M. Salamon

**Affiliations:** 1 Department of Diagnostic and Interventional Radiology and Nuclear Medicine, University Medical Center Hamburg-Eppendorf, Hamburg, Germany; 2 Department of Gynecology, University Medical Center Hamburg-Eppendorf, Hamburg, Germany; 3 Institute of Human Genetics, University of Ulm, Ulm, Germany; 4 Department of Neurology, University Medical Center Hamburg-Eppendorf, Hamburg, Germany; 5 Department of Obstetrics and Fetal Medicine, University Medical Center Hamburg-Eppendorf, Hamburg, Germany; 6 Department of Nuclear Medicine, Hannover Medical School, Hannover, Germany; University of Mississippi Medical Center, UNITED STATES

## Abstract

**Introduction:**

Patients with Neurofibromatosis type 1 (NF1) develop plexiform neurofibromas (PNF) and cutaneous neurofibromas. These tumors are a major cause of the patient’s morbidity and mortality. An influence of estrogen and progesterone on tumor growth has been suggested but reports on growth or malignant transformation of tumors during pregnancy remain anecdotal. The purpose of this study was to quantify growth of cutaneous and plexiform neurofibromas in NF1 patients during pregnancy, and to assess the onset of NF1 related symptoms.

**Material and methods:**

Retrospectively, 13 mothers with NF1 were included and compared to nullipara, nulligravida, age-matched women with NF1. All women received whole-body magnetic resonance imaging (MRI) before and after pregnancy or after a matched time period. Presence of plexiform and cutaneous neurofibromas was evaluated. PNF were subjected to semi-automated volumetry (MedX). The sum of the longest diameters (SLD) of representative cutaneous neurofibromas was determined for both groups. Clinical symptoms and subjective tumor growth were assessed.

**Results:**

PNF were identified in 12/26 women (46.2%). Follow up showed neither new PNF nor a significant difference in growth rate (median tumor-growth/year: pregnant group—0.38% (IQR -1.1–5.4%) vs control group 3.59% (IQR -2.1–5.5%; *P* = 0.69). Malignant transformation of PNF was not observed. There was a significant growth of cutaneous neurofibromas in both groups (median SLD increase: pregnant group 17mm; *P* = 0.0026 / control group 12mm; *P* = 0.0004) The difference in increase of SLD was not significant (*P* = 0.48). Singular cutaneous neurofibromas in the pregnant group displayed high levels of tumor growth (>20%/year). NF1-associated symptoms and subjective tumor growth were not significantly increased in pregnant patients.

**Conclusions:**

Growth of plexiform and cutaneous neurofibromas in pregnant patients is not significantly different compared to non-pregnant patients. Cutaneous neurofibromas show a significant increase in growth over time in both, pregnant and non-pregnant patients and NF1 related clinical symptoms do not significantly aggravate during the course of pregnancy.

## Introduction

Neurofibromatosis type 1 (NF1) is an autosomal dominantly inherited tumor predisposition syndrome with an incidence of 1 in 2500 newborns [1]. One hallmark feature of NF1 is the development of neurofibromas, e.g. cutaneous neurofibromas (NF) and plexiform neurofibromas (PNF) [2,3]. Large PNF can cause neurological, skeletal, vascular or obstructive abnormalities [4]. Growth of PNF inversely correlates with the age of patients, demonstrating significant growth of over 20% per year in patients under the age of 18 years [5]. Additionally, a large whole-body tumor volume is associated with an increased tumor growth [5]. Proposed risk factors for a large whole-body tumor volume are female gender and high numbers of subcutaneous Neurofibromas [6,7]. PNF can undergo transformation into malignant peripheral nerve sheath tumors (MPNST) in 8–13% of NF1 patients [8]. MPNST are the main cause of death in NF1 patients and a high whole-body tumor volume has been shown to be a risk factor for malignant transformation [9,10]. Therefore, NF1 patients repeatedly undergo whole-body magnetic resonance imaging (MRI) to evaluate tumor burden and malignant transformation [11,12]

It has been suggested that pregnancy in NF1 patients may cause an increase in volume of both cutaneous and subcutaneous neurofibromas [13,14]. Dugoff et al reported that 52.4% of women noticed growth of preexisting neurofibromas during pregnancy [13]. Anecdotal data indicate the possibility of an increase in size of PNFs during pregnancy with subsequent regression post-partum [15]. A potential cause for change in tumor volume during pregnancy is hormone dependent growth [16,17]. In vitro studies have shown that a majority of neurofibromas express progesterone receptors [18] and that exposure of tumor xenografts to estrogen or progesterone results in heterogeneous effects on proliferation and apoptosis [16,19]. Additionally, a significant growth of neurofibromas in patients with high-dose synthetic progesterone depot-contraceptives has been reported [20]. Other effects of pregnancy in NF1 patients are increased risk of gestational hypertension, preeclampsia, intrauterine growth restriction or preterm labor leading to an overall increased morbidity of NF1 patients compared to non-NF1 patients [13,21,22]. Exact evaluation and quantification of pregnancy-related growth of tumors and associated risks is desirable since a deeper understanding of the growth behavior of PNFs during pregnancy represents an unmet clinical need.

Therefore, the purpose of this study was to quantify growth of cutaneous and plexiform neurofibromas in NF1 patients during pregnancy, and to assess NF1 related clinical symptoms.

## Materials and methods

This retrospective, Health Insurance Portability and Accountability Act (HIPAA)-compliant study has been approved by the Ethical-Review Board of the “Ärztekammer Hamburg” and complied with the local data protection guidelines as well as the Declaration of Helsinki. Written informed consent was obtained from all participants.

### NF1 study population

We investigated 13 female NF1 patients before and after pregnancy (pregnant group; mean age at childbirth 25.3 years; SD ± 5.88 years; range 17–37 years) and an age-matched female control group (control group, mean age 24.6 years; SD ± 6.04 years; range 16–36 years) of nullipara, nulligravida women with NF1. Inclusion criteria were fulfillment of the National Institutes of Health (NIH) diagnostic criteria for NF1 [23], a history of pregnancy–or nulligravida in the control group—and availability of whole-body MRI scans performed before and after pregnancy or after a matched time period in the control group. Exclusion criteria were inability to undergo MRI or missing MRI examinations before or after pregnancy. The clinical follow up period for exclusion of malignant transformation of PNF was at least 18 months. Screening results for mutations of the neurofibromin gene were available for 11/13 patients of the pregnant group and for 12/13 patients of the control group **(S1 Table)**.

MR examinations were performed 35.3 months before (SD ± 22.7 months; range 11–55 months) and 36.9 months after (SD ± 36.9 months; range 6–108 months) pregnancy. Mean total observed time period in the pregnant group was 60.9 months (SD ± 60.9 months; range 24–123 months) compared to 51.8 months (SD 24.5 ± range 23–101 months) in the control group. The overall sum of patient years observed was 142.7.

### Image acquisition

Whole-body MRI was performed at 1.5T (Siemens Magnetom, Siemens Healthineers, Erlangen, Germany) between September 2014 and July 2017. MR-sequences consisted of T1w TSE coronal (TR 731ms; TE 11ms; FA 160°; Matrix 512x448; FOV 500x400mm; slice thickness 7mm; intersection gap 8.8mm), T2w TIRM coronal (TR 4000ms; TE 45ms; FA 130°; Matrix 384x384; FOV 499x399mm; slice thickness 7mm; intersection gap 8.8mm), T2w HASTE TIRM axial (TR 1200ms; TE 85ms, FA 148°; Matrix 384x384; FOV 450x450mm; slice thickness 8mm; intersection gap 10mm) and T2w TSE sagittal (TR 4600ms; TE 96ms; FA 160°; Matrix 512x504; FOV 350x320mm; slice thickness 3mm; intersection gap 3.3mm) sequences. Intravenous contrast material was not administered.

### Image analysis and volumetry

All examinations were evaluated regarding the presence of PNF or NF. PNF were defined on the basis of their characteristic appearance as signal intense polylobulated masses on T2-weighted sequences with growth along peripheral nerves [5,24]. Cutaneous or subcutaneous NF were defined as singular, round shaped lesions, localized cutaneously or subcutaneously, with random distribution, strongly hyperintense signal on T2-weighted and hypointense signal on T1-weighted sequences [24,25].

**Fig 1:**
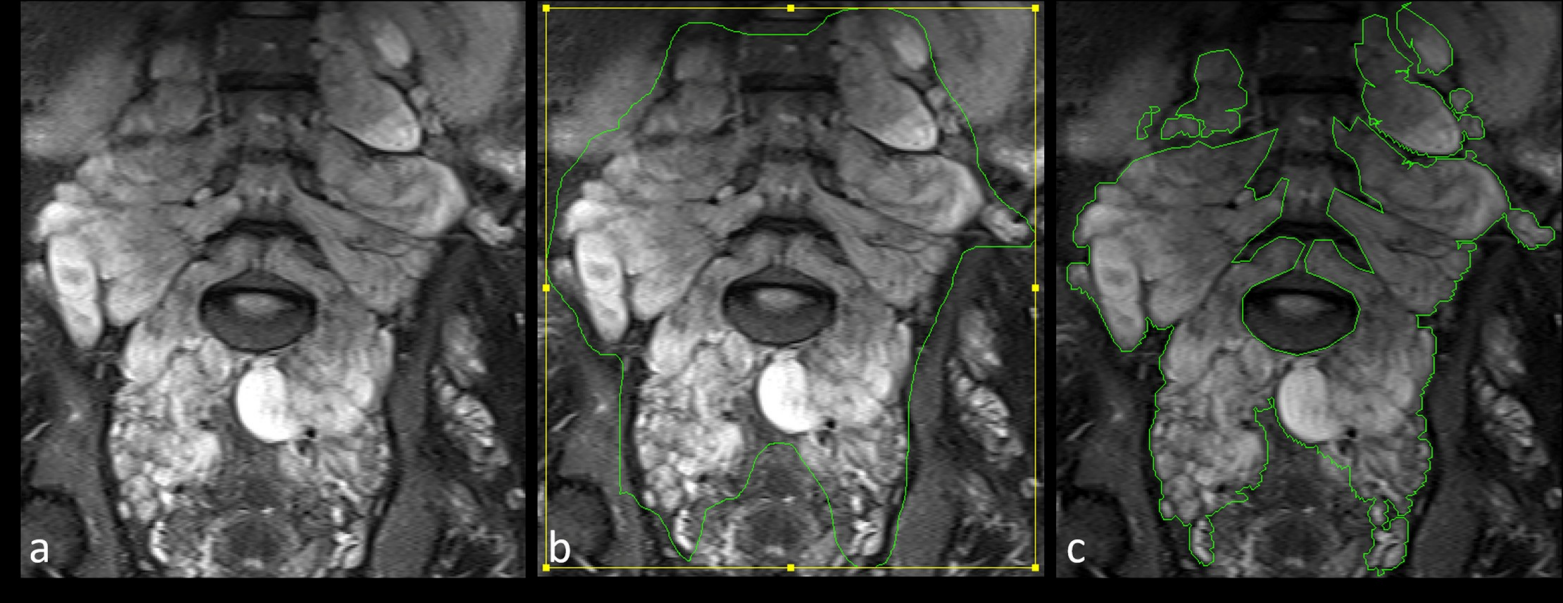
Illustration of the process of volumetry. Coronal T2 weighted STIR images of an 18-year-old patient with Neurofibromatosis type 1 from the nulliparous group. Note the diffuse T2 hyperintense plexiform neurofibromas growing along the lumbar and sacral nerves (a). Volumetry was performed by manually drawing regions of interest along tumor margins (b). MedX software then determined exact tumor margins to evaluate tumor volume (c).

Volumetry of PNFs was performed on T2-weigthed images using MedX software (v.3.42). MedX utilizes a heuristic-based semi-automated method for segmentation and measurement [26]. Differences of signal intensities in surrounding tissues (low intensity) and tumors (high intensity) are used to define tumor margins on each axial slice. Thereafter an automated volume calculation is performed. This volumetric assessment is sensitive and reproducible, yielding results similar to those of manual tracings [26] **(Fig 1)**. If automated border tracing was not feasible for a particular tumor, manual tracing was performed.

Diameters of 78 representative cutaneous or subcutaneous NF were measured manually and the sum of longest diameters (SLD) was calculated before and after pregnancy, or at initial examination and follow up, respectively. Tumor growth rates as determined by volumetry and changes in SLD were calculated for each patient and each tumor separately.

### Clinical information

Clinical symptoms of patients were assessed by questionnaire on a 4-point Likert scale in analogy to previously published questionnaires on NF1 [27] after completion of pregnancy/during follow up. The following items were included in both groups: Onset of NF1-associated musculoskeletal symptoms, subjective growth of neurofibromas and development of new cutaneous neurofibromas, history or development of MPNST. Patients in the pregnant group were additionally asked to answer questions regarding gestational hypertension, premature child delivery and tumor related complications during delivery. Items were rated as follows: 0 = none; 1 = mild; 2 = moderate; 3 = extensive.

### Statistical analysis

Continuous variables derived from tumor-volumetry are presented as median ± range. All variables were evaluated for normal distribution by the Shapiro-Wilk test. For pairwise comparison of continuous normally distributed data, a t-test for independent samples was applied. For not normally distributed data a Mann-Whitney U rank sum test was used. Paired Student’s t-tests were performed to determine significant differences in tumor volume or SLD over time. An increase in tumor volume over 20% per year was considered as significant tumor growth [5,26]. Categoric variables between different groups were compared by Fisher’s exact test for pairwise comparisons. Statistical analysis was performed using GraphPad Prism 5.0 for Windows (GraphPad Software Inc., La Jolla, CA). Statistical significance was assumed for *P*-values of less than 0.05.

## Results

### Distribution of neurofibromas

Cutaneous or subcutaneous neurofibromas were detected in 17 of 26 NF1-patients (65.4%), with eight out of 13 patients (61.5%) in the pregnant group and nine out of 13 patients (69.2%) in the control group. In the pregnant group, PNF were located in the head/neck (4/11; 36%), thorax (1/11; 9%), abdomen (2/11; 18%) and in the lower extremities (4/11; 36%). In the control group, PNF were located in the head/neck (2/9; 22%), thorax (1/9; 11%), abdomen (3/9; 33%) and in the lower extremities (3/9; 33%). During study interval and follow-up, none of the patients developed new PNFs. No patient developed a malignant peripheral nerve sheath tumor and no surgical resection of tumors was performed.

### Growth dynamics of plexiform neurofibromas during pregnancy

#### Per-patient analysis

In the total study population, the median whole-body PNF tumor burden was 108.1 ml (IQR 59.1–202.6 ml) at baseline and 124 ml (IQR 59.9–225.1 ml) at follow-up (*P* = 0.39).

**Fig 2:**
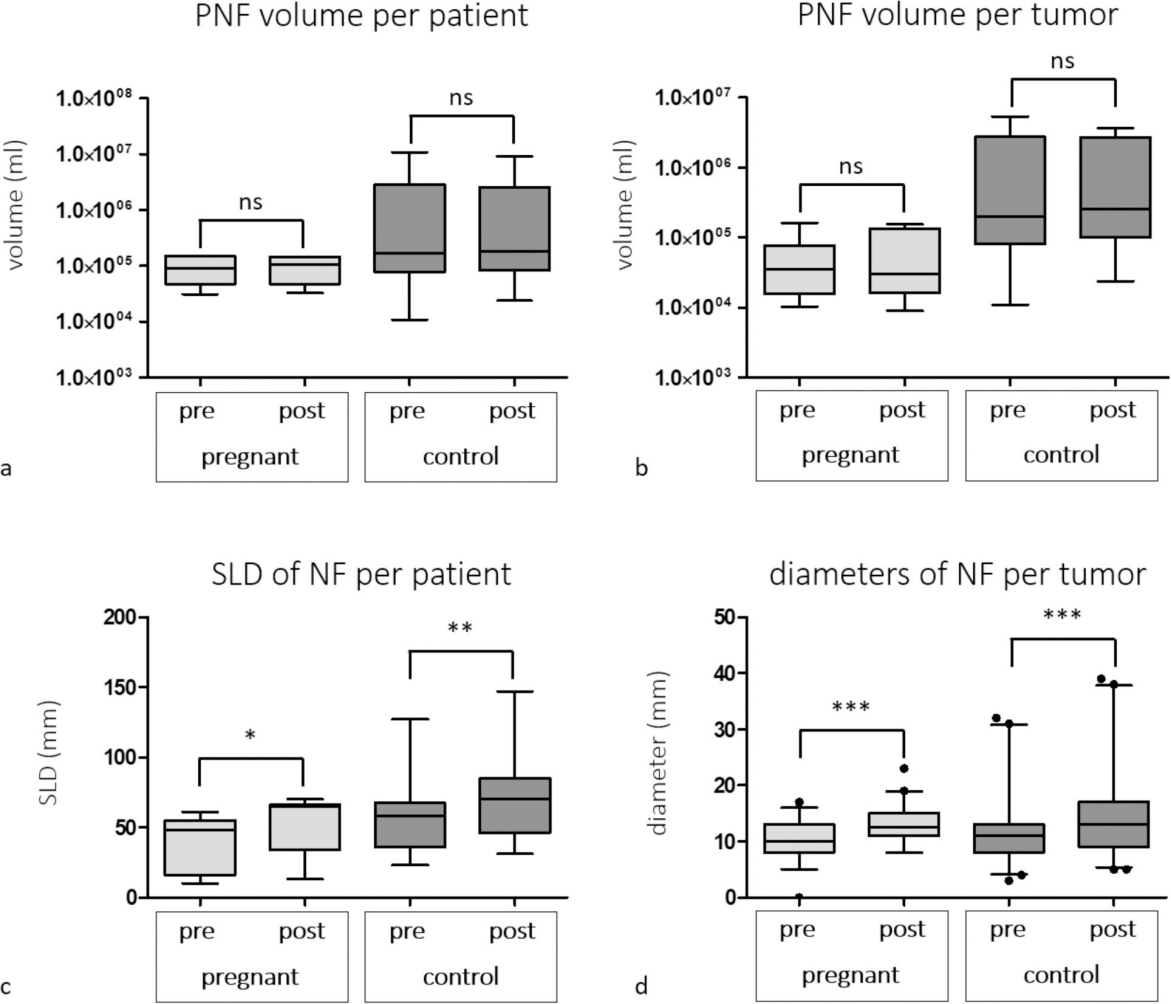
Box and whisker plots of volumes of plexiform neurofibromas (PNF) (a, b) and diameters of cutaneous neurofibromas (c, d) of pregnant patients and control patients on initial examination and follow up. Volumes are displayed on a per patient (a) and per tumor (b) basis, diameters are given as sum of longest diameters (SLD) per patient (c) and diameters of individual NF (d). There were no significant differences in tumor volume between initial examination and follow up for both, pregnant patients and control group (a, b). SLD and individual diameters of NF were significantly different between initial examination and follow up in both groups. NF: Neurofibroma; ns: not significant; PNF: Plexiform neurofibroma; SLD: sum of longest diameter; *: *P*<0.05; ** *P*<0.01; ***: *P*<0.0001.

The median whole-body PNF tumor burden in pregnant patients was 89.3 ml (IQR 47.6–150.1 ml) at baseline compared to 107.9 ml (IQR 47.5–146.4 ml) at follow-up (*P* = 0.67) **(Fig 2)**, whereas the median whole-body PNF tumor burden in controls was 167 ml (IQR 78.3–2911 ml) at baseline compared to 183.2 ml (IQR 82.8–2547 ml) at follow-up (*P* = 0.41) **(Fig 2)**. There was no significant difference between change in whole-body PNF tumor-burden over time between the pregnant group (overall median tumor-burden growth—1.7% (IQR -6–23%) and the control group (14.3%; IQR -12–22.2%) (*P* = 0.69) **(Table 1)**. Similarly, growth rates of pregnant patients (-0.38%; IQR -1.1–5.4%) and control group (3.59%; IQR -2.1–5.5%) were not significantly different (P = 0.69) **(Table 1)**. No patient demonstrated an increase in tumor-burden of ≥20% per year. Changes in tumor volume or diameters and growth rates for individual patients and tumors are given in **S2–S5 Tables.**

**Table 1:** Comparison of median tumor growth rates per year (%) from initial examination to follow up on a per patient and per tumor basis.

	growth rate per year	pregnant group	control group	*P*
**PNF**	**per patient**	-0.38% (-1.97–16.2)	3.59% (-3.37–6.83)	0.69
	**per tumor**	-0.6% (-4.1–11.4)	4.9% (-6.8–15.4)	0.32
**NF**	**per patient**	5.94% (2.57–11.97)	5.12% (3.32–7.41)	0.48
	**per tumor**	4.7% (0–40)	5.2% (0–32.1)	0.76

Given values indicate % tumor-growth per year; values in parentheses indicate range.

#### Per-lesion analysis

In the total study population, the median PNF lesion volume was 69.6 ml (IQR 21.6–190.3 ml) at baseline and 99.6 ml (IQR 24.1–227.3 ml) at follow-up (*P* = 0.80).

Lesions in pregnant patients demonstrated a median volume of 35.2 ml (IQR 15.8–77 ml) at baseline compared to 30.3 ml (IQR 16.5–133.1 ml) at follow-up (*P* = 0.64), whereas lesions in controls demonstrated a median volume of 199.4 ml (IQR 81.5–2734 ml) at baseline compared to 251.3 ml (IQR 99.6–2728 ml) at follow-up (*P* = 0.41). In the pregnant group 6/11 PNF (54.5%) increased in volume and 5/11 PNF (45.5%) decreased in volume over time, whereas in the control group 6/9 PNF (66.6%) increased in volume and 3/9 PNF (33.3%) decreased in volume (*P* = 0.67).

However, there was no significant difference between change in individual PNF volume over time between the pregnant group (median rate of tumor growth 0.3%; IQR -5.8–15.9%) and the control group (14.6%; IQR -12.1–42.4%) (*P* = 0.45) overall or on a per-year basis (median rate of tumor growth per-year; pregnant patients:-0.6%; IQR -3.4–0.9% vs. control patients: 4.9%; IQR -2.2–8.2%) (*P* = 0.22) **(Table 1)**. No individual PNF demonstrated an increase in volume of ≥20% per year **(S3 Table)**.

### Growth dynamics of cutaneous and subcutaneous neurofibromas during pregnancy

#### Per patient analysis

In the total study population, the median SLD was 48.5 mm (IQR 36–62.5mm) at baseline and 65 mm (IQR 45–74.5 mm) at follow up (*P*<0.0001). The median SLD of cutaneous neurofibromas in pregnant patients was 48 mm (IQR 16–55 mm) at baseline compared to 65 mm (IQR 34–66 mm) at follow up (*P* = 0.0026) **(Fig 2)**, whereas the median SLD in controls was 58 mm (IQR 36–67.5 mm) at baseline compared to 70 mm (IQR 46–85 mm) at follow-up (*P* = 0.0004) **(Fig 2)**.

While both groups demonstrated a significant increase in SLD over time, there was no significant difference between increase in SLD between the pregnant group 32% (IQR 25.4–37.1%) and the control group 22.2% (IQR 17–32.2%)(*P* = 0.24). The median increase in SLD per year was 5.9% (IQR 3.5–9.8%) in the pregnant group and 5.1% (IQR 3.9–7.4%) in the control group (*P* = 0.48) **(Table 1)**.

#### Per-lesion analysis

In the total study population, the median diameter of cutaneous neurofibromas was 10 mm (IQR 8–13 mm) at baseline and 13 mm (IQR 10–16 mm) at follow up (*P* = 0.0002). Lesions in pregnant patients demonstrated a median diameter of 10 mm (IQR 8–13 mm) at baseline compared to 12.5 mm (IQR 11–15 mm) at follow-up (*P*<0.0001) **(Fig 2)**. Lesions in the control group had a median diameter of 11 mm (IQR 8–13 mm) at baseline compared to 13 mm (IQR 9–17 mm) at follow-up (*P*<0.0001) **(Fig 2)**. There was no significant difference for increase in diameters over time between the pregnant group (median rate of increase in diameter 20% (IQR 0–57.1%) and the control group (22.2%; IQR 8.3–33.3%)(*P* = 0.98). The median rate of tumor growth per year was 4.7% (IQR 0.2–10%) in the pregnant group and 5.2% (IQR 1.6–8.5%) in the control group (*P* = 0.93). However, tumor diameters increased more than 20% per year in four neurofibromas in the pregnant group and in one neurofibroma in the control group (*P* = 0.19). Diameters of individual cutaneous neurofibromas are given in **S4 Table** and respective growth rates are displayed in S5 **& S6 Tables**.

### Evaluation of self-reported clinical symptoms and outcome of pregnancy

Seven of the 13 pregnant patients (53.8%), but no control patient (*P* = 0.0052) reported growth of pre-existing cutaneous neurofibromas and/or appearance of new neurofibromas **(Table 2)**. Three of 13 pregnant patients (23.1%), but no control patient (*P* = 0.22) experienced extensive musculoskeletal pain, whereas control patients reported only mild (46.2%) or moderate (23.1%) musculoskeletal pain. Three of 13 (23.1%) pregnant patients reported a history of gestational hypertension. None of the pregnant patients experienced pre-term delivery (i.e. <37 weeks of gestation) nor tumor-related complications during childbirth.

**Table 2:** Results of clinical survey in the pregnant group (n = 13) and control group (n = 13).

		pregnant group				control group		
	none	mild	extensive	severe	none	mild	extensive	severe
**Musculosceletal pain**	10/13 (76.9%)	0/13 (0%)	3/13 (23.1%)	0/13 (0%)	7/13 (53.8%)	6/13 (46.2%)	0/13 (0%)	0/13 (0%)
**subjective tumor growth**	0/13 (0%)	2/13 (15.4%)	4/13 (30.8%)	7/13 (53.8%)	7/13 (53.8%)	5/13 (38.5%)	1/13 (7.7%)	0/13 (0%)
**new tumors**	0/13 (0%)	4/13 (30.8%)	2/13 (15.4%)	7/13 (53.8%)	7/13 (53.8%)	4/13 (30.8%)	2/13 (15.4%)	0/13 (0%)
**MPNST**	13/13 (100%)	0/13 (0%)	0/13 (0%)	0/13 (0%)	13/13 (100%)	0/13 (0%)	0/13 (0%)	0/13 (0%)
**gestational hypertension**	10/13 (76.9%)	0/13 (0%)	0/13 (0%)	3/13 (23.1%)	n.a	n.a	n.a	n.a
**pre term delivery**	13/13 (100%)	0/13 (0%)	0/13 (0%)	0/13 (0%)	n.a	n.a	n.a	n.a

## Discussion

In this retrospective study, we investigated the effect of pregnancy on tumor burden in patients with NF1. Growth of plexiform and cutaneous neurofibromas in pregnant patients was not significantly different compared to non-pregnant patients. Some NF1 patients experience a subjective increase of NF1-related clinical symptoms and tumor growth during pregnancy. However, this increase of tumor growth or clinical symptoms was not significantly different to the control group.

PNFs were present in 46.2% of NF1-patients in our study, which is in line with previously published data [5,6,28]. Volumetry of PNF showed no significant growth of tumors in both, the pregnant group and the control group. Furthermore, no patient developed new PNF and no PNF underwent malignant transformation.

Volumes of PNF observed in our study are similar to the results of previous studies [5]. However, the tumor volume in our pregnant group (range 31.1–198.1ml) is small compared to others who identified volumes of up to 7553 ml in NF1 patients [28]. Additionally, the overall growth rate of PNF per year in our study was comparably low with no PNF showing significant growth of ≥ 20% per year. Other authors detected significant overall growth rates of PNF of 14.3% per year with maximum growth rates of up to 111% [5,28]. We did not identify development of new PNF in our patient collective within 142.7 patient years. This is in line with a previous study that found no development of new PNF within 273.0 patient years [5]. It also is in accordance with previously published reports that clinically apparent PNFs rarely develop in NF1 patients after early childhood [5]. With an overall lifetime risk of 8–15% for the development of MPNST, the observation that no PNF underwent malignant transformation in the investigated small patient group is expectable. Comparibly, Nguyen et al did not detect malignant transformation of a PNF in their patient collective with 273.0 patient years surveyed. Due to the partially long interval between MRI scans, a potentially short-termed effect of pregnancy on tumor growth (or volume, e.g. edema) cannot fully be excluded. However, data from a closely monitored patient collective with a median interval between MRI examinations of 3.9 months and a median number of MRI examinations of seven per patient suggested that plexiform neurofibromas do not exhibit erratic growth [28].

The heterogenous growth rate of PNF in our study and especially the slightly accentuated decrease in tumor volume in pregnant patients is an interesting observation. We found that more PNF in pregnant than in control patients showed a decrease in volume and that the overall growth rate of PNF in pregnant patients was reduced compared with the controls. This effect might be pregnancy related, since a hormone dependent decrease in volume of some but not all PNF has been described [16]. However, both abovementioned effects were not significant and larger studies are needed to confirm our observation. Additionally, other authors have described a decrease in tumor volume in non-pregnant NF1 patients in 35.5% of cases and negative growth rates of -3.6% [5,28]

Cutaneous neurofibromas showed significant growth in both groups, while the rate of growth between groups was not significantly different. The significant growth of cutaneous neurofibromas in our study is in accordance with previous reports, which indicate an increase in size of these tumors during pregnancy [13,14]. The only noteworthy difference between the two groups was a significant growth of four singular neurofibromas during pregnancy compared to significant growth of only one neurofibroma in the control group. This might indicate that singular neurofibromas can actually be affected by pregnancy, which is in accordance with published questionnaire based data and previously described heterogeneous responses of tumor growth to hormone exposure in vitro [13,16,19].

Clinical data as assessed by questionnaires showed an increase of subjective tumor growth, development of new neurofibromas, and NF1 associated musculoskeletal pain in some pregnant patients. However, volumetry of tumors did not confirm these subjective findings and statistical analysis did not confirm a significant increase of musculoskeletal pain in pregnant patients compared to the control group. This discrepancy between subjective tumor growth and volumetry might be caused by an increased awareness of pregnant patients regarding NF1 associated and physiological, pregnancy related bodily changes. However, the increase of clinical symptoms and tumor growth is similar to previous reports [13]. Incidence of gestational hypertension was higher than in previously published data, whereas incidence of pre-term labor was not [21]. We did not identify NF1 associated complications during child birth, contrary to other authors [21,22].

Our study has clinical implications. According to our data, an intensified monitoring of pregnant NF1 patients with regard to growth of PNF seems not to be necessary. Patients should be made aware that experienced growth of cutaneous neurofibromas during pregnancy is not significantly different from that in non-pregnant patients and that population based studies show an increase in pregnancy related morbidity compared to the non-NF1 population [21].

There are several limitations to our study. First of all, this study was performed retrospectively. Consecutively, the observed time period between baseline and follow-up MRI examinations as well as between MRI examinations and pregnancy varied between the included patients and was quite long in some cases. Therefore, a short-termed change in tumor growth would not have been detected. These limitations could be reduced in a prospective setting with controlled time periods between MRI examinations and pregnancy. Another limitation is the relatively small number of included pregnant NF1 patients, which is not only caused by the rarity of NF1 itself but also by the rather specific inclusion criteria. However, with regard to the specific inclusion criteria, the patient collective is comparable to other studies focusing on pregnancy in NF1 [29,30]. Another limitation is the lower volume of tumors in the pregnant group compared to the control group. A reason for this difference in tumor volume between the two groups might be an overall decreased chance of pregnancy in patients with severe manifestations of NF1 since a high level of visibility of NF1 can result in insecurity and sexual dissatisfaction [29]. One might reason that patients with higher tumors volumes might experience more or more severe complications during pregnancy. Additionally, it has been described that an overall high tumor volume correlates with an increased tumor growth. Therefore, one could expect an increased growth of tumors in the control group compared to the pregnant group in our study. However, this difference in median tumor volume between pregnant group and control group was not significant and mostly caused by one patient in the control group with a large tumor burden of 10,858.5ml.

In conclusion, our data show that growth of plexiform and cutaneous neurofibromas in pregnant patients is not significantly different compared to non-pregnant patients. Cutaneous neurofibromas show a significant increase in growth over time in both, pregnant and non-pregnant patients and NF1 related clinical symptoms do not significantly aggravate during the course of pregnancy. These findings should be confirmed in larger, multicenter studies and might be of use for patients with Neurofibromatosis type-1 who seek consultation before or during pregnancy.

## Supporting information

S1 Table Sequence changes identified in the NF1 gene of pregnant and control patients. Exons are numbered consecutively according to the NCBI nomenclature (1–57).
(DOCX)

S2 Table Volumes of individual plexiform neurofibromas and tumor volume per patient in pregnant and non-pregnant NF1 patients on baseline and follow-up examinations. Data are given in ml. NF1: Neurofibromatosis type 1—PNF: Plexiform neurofibroma.
(DOCX)

S3 Table Growth rates of plexiform neurofibromas in pregnant and non-pregnant NF1 patients. Growth rates are expressed as percentage of total volume of tumors measured on initial examination. Observational period is given in years and indicates the time between baseline and follow up examination. The difference in tumor volume is given in ml. PNF: Plexiform neurofibroma.
(DOCX)

S4 Table Diameters and sum of longest diameters (SLD) of cutaneous neurofibromas in pregnant and non-pregnant NF1 patients on baseline and follow up examinations. Data are given in mm. NF1: Neurofibromatosis type 1 –NF: neurofibroma.
(DOCX)

S5 Table Growth rates of cutaneous neurofibromas in pregnant NF1 patients. Growth rates are expressed as percentage of diameters of tumors measured on initial examination. Observational period is given in years and indicates the time between baseline and follow up examination. The difference in tumor diameter is given in mm.
(DOCX)

S6 Table Growth rates of cutaneous neurofibromas in non-pregnant NF1 patients. Growth rates are expressed as percentage of total volume of tumors measured on initial examination. Observational period is given in years and indicates the time between baseline and follow up examination. The difference in tumor diameter is given in mm.
(DOCX)
