# Supplementary material for: The effect of pregnancy on growth-dynamics of neurofibromas in Neurofibromatosis type 1
Source: PLoS One. 2020 Apr 28;15(4):e0232031. doi: 10.1371/journal.pone.0232031 (PMC7188260; doi:10.1371/journal.pone.0232031)
Supplement: S1 Table — Exons are numbered consecutively according to the NCBI nomenclature (1–57). (DOCX) [file pone.0232031.s001.docx]

**Supplementary Table S1:** Sequence changes identified in the NF-1 gene of pregnant and control patients.

| **Patient** | **Group** | **Variant type** | **Location** | **DNA change** | **child affected** |
| --- | --- | --- | --- | --- | --- |
| **#1** | pregnant | deletion | whole gene | type 2 deletion | no |
| **#2** | pregnant | not performed | - | - | na |
| **#3** | pregnant | frameshift | E 21 | c.2581_2582 del GC | yes |
| **#4** | pregnant | splice | E 15 | c.1642-8 A-G | yes |
| **#5** | pregnant | splice | E 23 | c.3113 G>C | no |
| **#6** | pregnant | missense | E 17 | c.1885 G>A | na |
| **#7** | pregnant | frameshift | E 23 | c.3044_3045 del TG | no |
| **#8** | pregnant | nonsense | E 44 | c.6709 C>T | yes |
| **#9** | pregnant | frameshift | E 54 | c.7925 ins T | yes |
| **#10** | pregnant | splice | E 22 | c.2851_2852 del A | yes |
| **#11** | pregnant | not performed | - | - | na |
| **#12** | pregnant | splice | E 32 | c.4270_4272 A>G | na |
| **#13** | pregnant | frameshift | E 22 | c.2900_2901 del TA | yes |
| **#1** | control | no proof | - | - | - |
| **#2** | control | splice | E 52 | c.7676-2 A>G | - |
| **#3** | control | no proof | - | - | - |
| **#4** | control | no proof | - | - | - |
| **#5** | control | frameshift | E 17 | c.1907CTAG>ACA | - |
| **#6** | control | no proof | - | - | - |
| **#7** | control | no proof | - | - | - |
| **#8** | control | deletion | whole gene | type 1 deletion | - |
| **#9** | control | missense | E 47 | c.7052 C>T | - |
| **#10** | control | splice | E 23 | c.3113 G>C | - |
| **#11** | control | missense | E 27 | c.3662 T>G | - |
| **#12** | control | not performed | - | - | - |
| **#13** | control | splice | E 30 | c.3975-2 A>G | - |

Exons are numbered consecutively according to the NCBI nomenclature (1-57).
