# Supplementary material for: The effect of pregnancy on growth-dynamics of neurofibromas in Neurofibromatosis type 1
Source: PLoS One. 2020 Apr 28;15(4):e0232031. doi: 10.1371/journal.pone.0232031 (PMC7188260; doi:10.1371/journal.pone.0232031)
Supplement: S2 Table — Data are given in ml. NF1: Neurofibromatosis type 1—PNF: Plexiform neurofibroma. (DOCX) [file pone.0232031.s002.docx]

**Supplementary Table S2:** Volumes of individual plexiform neurofibromas and tumor volume per patient in pregnant and non-pregnant NF-1 patients on baseline and follow-up examinations.

| **pregnant group** | | | | | | | | |
| --- | --- | --- | --- | --- | --- | --- | --- | --- |
| **baseline** | | | | | **follow up** | | | |
| **Patient** | **PNF 1** | **PNF 2** | **PNF 3** | **total volume** | **PNF 1** | **PNF 2** | **PNF 3** | **total volume** |
| **#2** | 14.3 | 38.8 | - | **53.1** | 13.5 | 41.9 | - | **55.4** |
| **#7** | 20.8 | 10.3 | - | **31.1** | 24.1 | 9 | - | **33.1** |
| **#8** | 152.6 | - | - | **152.6** | 146.4 | - | - | **146.4** |
| **#9** | 162.9 | 35.2 | - | **198.1** | 155.3 | 30.3 | - | **185.6** |
| **#10** | 24.1 | 15.8 | 61.7 | **39.9** | 24.1 | 16.5 | 49.1 | **89.7** |
| **#11** | 77 | - | - | **77** | 133.1 | - | - | **133.1** |
| **control group** | | | | | | | | |
| **baseline** | | | | | **follow up** | | | |
| **Patient** | **PNF 1** | **PNF 2** | **PNF 3** | **total volume** | **PNF 1** | **PNF 2** | **PNF 3** | **total volume** |
| **#1** | 62.2 | 199.4 | - | **261.6** | 96.8 | 257.7 | - | **354.5** |
| **#2** | 219.3 | - | - | **219.3** | 251.3 | - | - | **251.3** |
| **#3** | 100.8 | - | - | **100.8** | 115.0 | - | - | **115.0** |
| **#8** | 114.6 | - | - | **114.6** | 102.3 | - | - | **102.3** |
| **#11** | 10.9 | - | - | **10.9** | 23.8 | - | - | **23.8** |
| **#13** | 2403.4 | 3063.6 | 5391.5 | **10858.5** | 2769.6 | 2686.2 | 3668.9 | **9125** |

Data are given in ml.

NF-1: Neurofibromatosis type 1 - PNF: Plexiform neurofibroma
