# Supplementary material for: The effect of pregnancy on growth-dynamics of neurofibromas in Neurofibromatosis type 1
Source: PLoS One. 2020 Apr 28;15(4):e0232031. doi: 10.1371/journal.pone.0232031 (PMC7188260; doi:10.1371/journal.pone.0232031)
Supplement: S3 Table — Growth rates are expressed as percentage of total volume of tumors measured on initial examination. Observational period is given in years and indicates the time between baseline and follow up examination. The difference in tumor volume is given in ml. PNF: Plexiform neurofibroma. (DOCX) [file pone.0232031.s003.docx]

**Supplementary Table S3:** Growth rates of plexiform neurofibromas in pregnant and non-pregnant NF-1 patients.

| **pregnant group** | | | | | |
| --- | --- | --- | --- | --- | --- |
| **Patient** | **PNF** | **growth rate (%)** | **period of observation (years)** | **growth rate / year (%)** | **difference in volume (ml)** |
| **#2** | # 1 | - 5.8 | 9.08 | - 0.64 | - 0.8 |
|  | # 2 | 7.9 | 9.08 | 0.87 | 3.1 |
| **#7** | # 1 | 15.9 | 3.5 | 4.54 | 3. |
|  | # 2 | - 12.9 | 3.5 | -3.69 | - 1.3 |
| **#8** | # 1 | - 4.7 | 4.75 | - 0.86 | - 6.2 |
| **#9** | # 1 | - 4.1 | 3.17 | - 1.48 | - 7.6 |
|  | # 2 | - 13.1 | 3.17 | - 4.13 | - 4.8 |
| **#10** | # 1 | 0.3 | 6.0 | 0.05 | 0.1 |
|  | # 2 | 4.1 | 6.0 | 0.68 | 0.6 |
|  | #3 | - 20.5 | 6.0 | - 3.42 | - 12.7 |
| **#11** | #1 | 51.3 | 4.5 | 11.4 | 56.1 |
| **control group** | | | | | |
| **Patient** | **PNF** | **growth rate (%)** | **period of observation (years)** | **growth rate / year (%)** | **difference in volume (ml)** |
| **#1** | # 1 | 55.5 | 5.2 | 10.68 | 34.6 |
|  | # 2 | 29.2 | 5.2 | 5.62 | 58.3 |
| **#2** | # 1 | 14.6 | 3.0 | 4.87 | 32 |
| **#3** | # 1 | 14.1 | 2.8 | 5.03 | 14.2 |
| **#8** | # 1 | - 11.7 | 6.25 | - 1.87 | - 12.2 |
| **#11** | # 1 | 117.8 | 7.67 | 15.35 | 12.9 |
| **#13** | # 1 | 15.23 | 4.75 | 3.21 | 366.2 |
|  | # 2 | - 12.4 | 4.75 | - 2.61 | - 377.3 |
|  | # 3 | - 32.1 | 4.75 | - 6.75 | - 1722 |

Growth rates are expressed as percentage of total volume of tumors measured on initial examination. Observational period is given in years and indicates the time between baseline and follow up examination. The difference in tumor volume is given in ml.

PNF: Plexiform neurofibroma
