# Supplementary material for: The effect of pregnancy on growth-dynamics of neurofibromas in Neurofibromatosis type 1
Source: PLoS One. 2020 Apr 28;15(4):e0232031. doi: 10.1371/journal.pone.0232031 (PMC7188260; doi:10.1371/journal.pone.0232031)
Supplement: S4 Table — Data are given in mm. NF1: Neurofibromatosis type 1 –NF: neurofibroma. (DOCX) [file pone.0232031.s004.docx]

**Supplementary Table S4:** Diameters and sum of longest diameters (SLD) of cutaneous neurofibromas in pregnant and non-pregnant NF-1 patients on baseline and follow up examinations.

| **pregnant group** | | | | | | | | | | | | |
| --- | --- | --- | --- | --- | --- | --- | --- | --- | --- | --- | --- | --- |
| **baseline** | | | | | | | **follow up** | | | | | |
| **Patient** | **NF 1** | **NF 2** | **NF 3** | **NF 4** | **NF 5** | **SLD** | **NF 1** | **NF 2** | **NF 3** | **NF 4** | **NF 5** | **SLD** |
| **#2** | 0 | 12 | 16 | 14 | 8 | **55** | 9 | 17 | 16 | 15 | 11 | **70** |
| **#4** | 10 | 11 | 13 | 12 | 15 | **61** | 12 | 11 | 12 | 12 | 18 | **65** |
| **#5** | 14 | 18 | 8 | 9 | 13 | **62** | 15 | 18 | 8 | 9 | 13 | **63** |
| **#6** | 6 | 9 | 8 | 10 | 9 | **42** | 8 | 10 | 8 | 11 | 15 | **52** |
| **#7** | 8 | 10 | 6 | 11 | 13 | **48** | 10 | 17 | 11 | 14 | 14 | **66** |
| **#8** | 7 | 15 | 11 | 9 | 7 | **49** | 14 | 19 | 11 | 10 | 11 | **65** |
| **#10** | 6 | 5 | 11 | 7 | 5 | **34** | 13 | 17 | 13 | 11 | 10 | **64** |
| **#11** | 9 | 13 | 16 | 10 | 10 | **58** | 15 | 16 | 23 | 12 | 13 | **79** |
| **control group** | | | | | | | | | | | | |
| **baseline** | | | | | | | **follow up** | | | | | |
| **Patient** | **NF 1** | **NF 2** | **NF 3** | **NF 4** | **NF 5** | **SLD** | **NF 1** | **NF 2** | **NF 3** | **NF 4** | **NF 5** | **SLD** |
| **#1** | 9 | 11 | 6 | 7 | 3 | **36** | 7 | 13 | 12 | 8 | 8 | **48** |
| **#2** | 13 | 10 | 16 | 11 | 13 | **63** | 16 | 12 | 19 | 13 | 17 | **77** |
| **#3** | 12 | 12 | 13 | 10 | 11 | **58** | 18 | 11 | 16 | 10 | 15 | **70** |
| **#5** | 16 | 19 | 8 | 9 | 13 | **65** | 37 | 23 | 9 | 11 | 13 | **93** |
| **#6** | 6 | 7 | 11 | 5 | 9 | **38** | 8 | 9 | 16 | 5 | 11 | **49** |
| **#7** | 4 | 12 | 7 | 8 | 5 | **36** | 5 | 13 | 7 | 10 | 9 | **44** |
| **#8** | 12 | 31 | 32 | 22 | 30 | **127** | 17 | 31 | 39 | 22 | 38 | **147** |
| **#10** | 9 | 12 | 14 | 25 | 10 | **10** | 10 | 13 | 19 | 23 | 11 | **76** |
| **#11** | 7 | 8 | 8 | - | - | **23** | 10 | 13 | 8 | - | - | **31** |

Data are given in mm.

NF-1: Neurofibromatosis type 1 – NF: neurofibroma
