# Supplementary material for: The effect of pregnancy on growth-dynamics of neurofibromas in Neurofibromatosis type 1
Source: PLoS One. 2020 Apr 28;15(4):e0232031. doi: 10.1371/journal.pone.0232031 (PMC7188260; doi:10.1371/journal.pone.0232031)
Supplement: S6 Table — Growth rates are expressed as percentage of total volume of tumors measured on initial examination. Observational period is given in years and indicates the time between baseline and follow up examination. The difference in tumor diameter is given in mm. (DOCX) [file pone.0232031.s006.docx]

**Supplementary Table S6:** Growth rates of cutaneous neurofibromas in non-pregnant NF-1 patients.

| **control group** | | | | | |
| --- | --- | --- | --- | --- | --- |
| **Patient** | **NF** | **growth rate over time (%)** | **period of observation** | **growth rate / year (%)** | **difference in tumor diameter (mm)** |
| **#1** | # 1 | -33 | 5.2 | 5.16 | - 2 |
|  | # 2 | 29.2 |  | 3.5 | 2 |
|  | # 3 | 100 |  | 19.23 | 6 |
|  | # 4 | 14.3 |  | 27.8 | 7 |
|  | # 5 | 166.7 |  | 2.75 | 5 |
| **#2** | # 1 | 23.1 | 3.0 | 7.7 | 3 |
|  | # 2 | 20 |  | 6.7 | 2 |
|  | # 3 | 18.8 |  | 6.3 | 2 |
|  | # 4 | 18.2 |  | 6.1 | 0 |
|  | # 5 | 30.8 |  | 10.3 | n/a |
| **#3** | # 1 | 50 | 2.8 | 17.9 | 6 |
|  | # 2 | - 8.4 |  | -3 | - 1 |
|  | # 3 | 23.1 |  | 8.24 | 3 |
|  | # 4 | 0 |  | 0 | 3 |
|  | # 5 | 36.4 |  | 36.4 | 4 |
| **#5** | # 1 | 131.3 | 8.42 | 15.6 | 21 |
|  | # 2 | 21.1 |  | 2.5 | 4 |
|  | # 3 | 12.5 |  | 1.48 | 1 |
|  | # 4 | 22.22 |  | 2.64 | 2 |
|  | # 5 | 0 |  | 0 | 0 |
| **#6** | # 1 | 33.33 | 3.92 | 8.5 | 2 |
|  | # 2 | 28.57 |  | 7.3 | 2 |
|  | # 3 | 45.45 |  | 11.6 | 5 |
|  | # 4 | 0 |  | 0 | 0 |
|  | # 5 | 22.22 |  | 5.7 | 1 |
| **#7** | # 1 | 25 | 5.08 | 4.92 | 1 |
|  | # 2 | 8.3 |  | 1.64 | 1 |
|  | # 3 | 0 |  | 0 | 0 |
|  | # 4 | 25 |  | 4.92 | 2 |
|  | # 5 | 80 |  | 15.8 | 4 |
| **#8** | # 1 | 41.7 | 4.75 | 8.8 | 5 |
|  | # 2 | 0 |  | 0 | 0 |
|  | # 3 | 21.9 |  | 4.67 | 7 |
|  | # 4 | 0 |  | 0 | 0 |
|  | # 5 | 26.7 |  | 5.61 | 8 |
| **#10** | # 1 | 11.1 | 2.5 | 4.4 | 1 |
|  | # 2 | 8.3 |  | 3.3 | 1 |
|  | # 3 | 35.71 |  | 14.3 | 5 |
|  | # 4 | - 8 |  | - 3.2 | - 2 |
|  | # 5 | 10 |  | 0 | 1 |
| **#11** | # 1 | 42.86 | 7.67 | 5.59 | 3 |
|  | # 2 | 62.5 |  | 8.15 | 6 |
|  | # 3 | 0 |  | 0 | 0 |

Growth rates are expressed as percentage of total volume of tumors measured on initial examination. Observational period is given in years and indicates the time between baseline and follow up examination. The difference in tumor diameter is given in mm.
